# Medical Miscellanies

**Published:** 1816-02

**Authors:** 


### PART IV.

### MEDICAL MISCELLANIES.

### ELECTRICITY.

THE most convenient apparatus for Medico-Electrical purposes consists of a circular glass plate, measuring from 18 to 28 inches in diameter, and revolving between two pairs of dry leathern cushions, a conductor, Leyden jar with a moderately sized cylinder, and medical electrometer. With a machine thus constructed, and its appurtenances, an insulating stool or chair, a brass ball or wooden-pointed director mounted in glass and having a wooden handle, another director inserted in wood, and some small wire or chain, electricity may be applied to the human body in a variety of ways, so as to be augmented or diminished in every possible degree, and to correspond with every curative intention.

When it is merely required to electrify a part of the body, one end of a smooth wire is to be affixed to the conductor or electrometer, and its other extremity attached to the glass-mounted director with a pointed extremity, from which the fluid is readily poured upon the part affected. When the sparks are necessary, a ball is to be substituted for the point. When the shock is wanted, the circuit of the Leyden jar is to be made, which consists in causing the patient to become a conducting medium between the outside of the jar and the electrometer; which last is placed at a convenient distance from the jar, to attract the discharge of its contents, when collected in sufficient quantity by the revolutions of the plate.

The electrometer enables us to regulate at pleasure the strength of the shock; and the directors, by enclosing the patient so as to make him a link of the connecting chain, assist us in transmitting the electrical fluid through any local morbid affection, without disturbing the general system. The principal distinctions of electrical application are the following:

1st. *The Aura*, or invisible electricity, which is by some denominated simple electrization, and consists in the electric fluid being drawn, by means of a director communicating with the earth, from the patient, whilst he is insulated upon a glass pedestal or other non-conductor; as is usually practised in cases of amaurosis or gutta serena, gout, nervous affections, cephalalgia, &c.

2d. *The Radii.* When the fluid is projected from a point in form of a brush, and in colour of a beautiful cærulian tint, or when attracted by a similar contrivance from the patient saturated with the fluid and in a state of insulation: as we customarily administer it in ophthalmia, opacities of the cornea transparens, obstructions of the glandulæ lachrymales, and Meibomiæ, ductus nasalis, and puncta lachrymalia; cutaneous diseases of the face, local inflammations, acute rheumatism, and when an increased serous discharge is required from a blistered surface.

3d. *The Spark.* When many of these radii are concentrated, and break over a ball, in a stellated vermilion appearance, upon the surface of the body, or any diseased organ; or drawn therefrom, when insulated, by an obtuse point closely applied: as is practised in intermittents and hectic fevers, chronic rheumatism, deranged functions of the liver, stomach, and other viscera; suppressed secretions, as of the perspiration in diabetes; extravasations, of the serosity of the blood in anasarca; sprains and bruises; sensorial derangements of a temporary nature, as hysteria, epilepsia, nymphomania, or furor uterinus, relaxations of parts, as in paralysis partialis, and scrophulous enlargements of secretory organs; muscular contractions, rigidity and immobility of articulations, spasmodic affections, and painful suppression or obstruction of accustomed evacuations, as mænorrhagia difficilis, constipation of the bowels, &c.

4th. *The globe or shock*, which differs from the spark only in being much stronger, by the accumulation of a larger quantity of the electric fluid in the jar, which is discharged according to any required force, by a judicious arrangement of the electrometer. This form of electricity assumes a spherical or globular figure, and appears of a brilliant scarlet colour; it is administered as a stronger, and, on some occasions, a more efficacious method than the foregoing. In many cases, it is employed either to confirm a cure partially effected by sparks, or to act vigorously upon parts to which the latter has been fruitlessly applied; as in paralysis hemiphlegica, paralysis venenata, cases of suspended animation from hanging or drowning, schirrosities of the mammæ and other glandular parts, induratio testis, hydro-sarcocele, ganglia, deficiency of callus in the divided extremities of fractured bones, hydrocephalus, hydrops articuli, and chronic cases of cerebral, spinal, renal, uterine, ovarian, splenic, enteritic, and spermatic diseases: irritation of particular nerves, as the infra orbital branch of the ramus ocularis nervi trigemini, (which, when diseased, constitutes the faciei nervus morborum crucians of Fothergill) obstinate degrees of amenorrhœa, and above all, scrophulous diseases of joints, as white swelling, &c.

BENJAMIN GOLDING.

No. 13, Leicester Place, Leicester Square.

---

*Case of Ligature of the common carotid Artery, in consequence of a Gun-shot Wound.*—In the spring of 1814, a Russian soldier was struck by a musket-ball; which, after fracturing the condyle and coronoid process of the lower jaw on the right side, projected beneath the integuments of the neck about the spine of the third cervical vertebra: from whence it was extracted by slight incision. On the eighth day of the wound, the eschar separated; and considerable effusion of arterial blood took place. Compression failed to arrest the hæmorrhage. One or other branch of the common carotid artery was evidently wounded. The following is the account given by Dr. Giroud, a French surgeon, of the operation which he performed.

"After having divided the integuments and made a partial incision of the parotid gland, an attempt to stop the hæmorrhage by an introduction of the fingers into the wound, failed: it was completely inundated by the jet of blood. The incision was now extended within an inch of the clavicle, and the common carotid artery exposed. This vessel, after being separated from the internal jugular vein, eighth nerve, and surrounding cellular membrane, was tied; *a piece of agaric being included in the ligature, in order to obviate premature division of the arterial coats, and thus prevent secondary hæmorrhage.* No obstacle was encountered in the operation, except the division of the thyroid vein; which was immediately secured. A grooved director, flexible probe, and curved needle, were the instruments employed in this operation."

"Every thing went on favorably until the seventh day from the operation. Neither intellectual faculties, nor the functions of the organs of sense, had been at all disturbed. But, at that period, stupor, confusion of ideas, restlessness, small unsteady pulse; discoloration of the visage, and loss of strength, came on; followed, in the evening, by a violent paroxysm of fever. On the eighth day, a considerable hæmorrhage took place at three several times: it oozed from the whole surface of the wound; and was, at last, suppressed by compress and bandage. The fever recurred. On the ninth day, the man died. The common carotid artery was found, on examination, to have been tied an inch and a half below its division. The arterial coats were entire; the internal, slightly inflamed: a coagulum of blood, tolerably thick and firm, plugged each extremity of the vessel adjacent to the ligature. The wound of the external carotid artery had been inflicted some lines above the division of the common trunk into its two primary branches."

Dr. Giroud also cursorily mentions a case in which, "about the same time, a French surgeon, at the military hospital of Toulouse, passed a ligature *round the trunks of the common carotid artery and internal jugular vein.* Both these vessels had been wounded by a musket-shot which struck the subject of the operation, a soldier, about the angle of the jaw. Until the sixth day from the application of the ligature, nothing unfavorable had occurred." The result of the operation, however, had not been communicated to the public so lately as last July. *Journal géneral de médecine, &c. par Sedillot.*

The operation of Dr. Giroud requires no comment. It conveys a tolerably correct idea of French practice in the surgical treatment of aneurism and wounded arteries. Surely the time is not far distant when their prejudices, respecting the application of the ligature to arteries, will be dissipated by the light which the intrepidity and genius of our countrymen have thrown upon it. To some able French surgeon we earnestly recommend a translation of the work of Mr. Hodgson, on the diseases of arteries, into the French language.

*Apparent Death from Fumes of Seacoal.*—During the severe winter of 1813, while our fleet lay frozen up in the Scheldt, blockading Walcheren, one of the boys belonging to a frigate near Zeirickzee, shut himself up in a small cabin, in which he was attending a swinging stove, and a short time after was found lying on the deck of the cabin in a state of insensibility. The symptoms were, a complete suspension of the powers of voluntary motion, and of sensation; subsultus tendinum; respiration suspended; slight vibratory motion about the heart; no pulsation at the wrists; extremities cold; trunk moderately warm; face almost of its natural colour; eyes not at all suffused; pupils appeared to contract on the approach of a lighted candle; limbs perfectly flexible; a little rigidity of the jaw; mouth partly open. He was perfectly insensible to severe pinching, or to the effluvium of carbonate of ammonia applied to the nose. He was recovered in about half an hour, by the unremitting use of those means recommended by the Humane Society; but particularly friction, bottles of warm water to the epigastrium, feet and hands, &c. When about to administer a stimulating enema, he breathed, and uttered a faint ejaculation, on his name being loudly pronounced in his ear. He now fetched four deep inspirations, at intervals of full five minutes, during which time his recovery was frequently doubtful. Nothing could be got down his throat all this time. He completely recovered.

*Cardiac Affection.*—A young lady, 22 years of age, after a severe cold and cough, became affected with a palpitation of the heart, and a swelling on each side of the neck, as large as a goose's egg, accompanied with dyspnœa on the slightest exertion, and inability to lie in bed, except when the head was much raised. These symptoms had continued for six months when the Reporter saw her. On first opening the breast and exposing her neck, the two tumors appeared to pulsate, both to the eye and the hand, like immense carotid aneurisms; and a strong pulsation was observable down the left side to the left hypochondriac region, and also at the epigastrium. She was extremely nervous, the least noise or exercise throwing her into a state of violent agitation and alarm, accompanied with such distressing dyspnœa and palpitation of the heart, that she thought her chest would burst. On a minute examination of the tumors in the neck, the arterial pulsation was so unequivocal that scarcely a doubt could be entertained of their aneurismal origin; at least, the Reporter could not decide that they were *not* so. That great functional, if not structural, derangement had also taken place in the heart was quite manifest. The lady had that very morning been taking bark and port wine, for her apparent debility, under the direction of a medical friend, and had been using discutient applications to the tumors without success. The young lady was tall, extremely plethoric, and the eyes had a prominence as though they were about to start from their sockets. Her pulse varied every moment with her state of mind; her bowels were constipated; her appetite not impaired, and by no means restricted by her medical adviser.

She was ordered to be bled immediately; and this operation to be often repeated afterwards, if necessary; to abstain *in toto* from animal food, and every kind of drink but water; to make use of no exertion, and to keep her bowels very open with the following pills:

℞. Ex. hyoscyami ℈j. Ex. colocynth. comp. ʒj. Submur. hydrarg. gr. x. Pulv. digitalis ℈j. Misce ft. et divide in Pil. No. xxx quarum capiat unam vel duas mane ac vesperi.

The young lady returned to the country, and as the Reporter heard nothing of her for seven months, he concluded she was very ill, perhaps dead. He was agreeably disappointed a few days ago, to meet her in the street, apparently in perfect health!

Anxious to know what had happened in the interval, he accompanied her to her lodgings, and was informed, that she had obeyed the injunctions given her; had been twice or thrice bled; abstained from animal food, &c.; and now found herself comparatively well, there being only a moderate palpitation at the heart on walking fast, or going up stairs, and the tumors were greatly reduced in size. The reporter examined the tumors carefully, and found them to be enlargements of the two lobes of the thyroid gland. They were so reduced in size now, that they were moveable, and their structure easily made out, but a very trifling *communicated* pulsation was perceptible in them. Her eyes were no longer prominent; her dyspnœa and palpitatio, except on considerable exertion, were no longer distressing; in short, the whole tenor of her formerly alarming symptoms was changed into comparative health.

This case, while it shews the erroneous and dangerous view of it which was taken during the bark and wine system, may serve as a caution against too desponding a prognostic under apparently desperate states of the function or structure of the heart.

*Case of Malformation of the Urinary and Genital Organs, by Dr. Palmer.*—The child, who formed the subject of this examination, was born at the full term of pregnancy; of the middle size, healthy, and in general well formed. But the deficiency of an anus, and peculiarity in the formation of the genitals were soon detected. At first, the child ate, drank, and slept in a natural manner; but appearances of restlessness and distress came on after a few hours; and the child died on the second day. No clear account of the symptoms could be gleaned, as they had not been watched by a medical attendant. The child had displayed signs of sickness, but rejected nothing. The body was examined on the following morning.—*External appearances.* Abdomen tense and swollen, as though from a distended state of the bladder and bowels. The buttocks well formed; the sulcus, separating them, of the natural depth: no appearance of anus, or prominence or depression marking its natural site : the genitals, at first glance, resembling those of a male child ; but a large projecting, mis-shapen, imperforate clitoris, with its prepuce, was, on closer examination, found to have been mistaken for a penis; the prominent and apparently-united labia, for a scrotum. On separating the labia, the regularly formed nymphæ were discovered; and, in a sort of depression between them, and about three lines below the insertion of the clitoris, a small orifice, encircled by a spongy prominent margin. It merely admitted the head of a common silver probe to the depth of about the third of an inch ; and was clearly the opening of an imperfect urethra. The delicate membrane of the nymphæ passed between these two bodies, and obliterated every trace of vagina.— *Internal appearances.* The bladder distended with healthy urine, was situated naturally behind the pubis : the ureters entered the organ in the usual place and manner; but no route for the evacuation of its contents could be found. The colon, after reaching the vicinity of the left kidney, began, as it descended, to form a sigmoid flexure ; but, before it reached the concavity of the left ilium, made a sudden turn to the right; and, crossing the psoas muscle, arrived at the projection of the sacrum, where it terminated abruptly, *without at all entering the pelvis.* The intestine was very firmly bound down by a slip of thin but strong membrane to the sacral projection. Directly behind the bladder, and so closely attached to it as not to be separable by the most cautious dissection, was situated a pyriform, or rather triangular, membranous cyst, containing apparently more than two ounces of a turbid whey-like fluid. It was supported, independently of its attachment to the bladder, and partly inclosed, much in the same manner as the uterus commonly is, by a transverse production of peritoneum. Nothing like the ovaria or Fallopian tubes could be found ; but the spermatic vessels, unusually large and injected with blood, were clearly traced to the two superior obtuse angles of the cyst, where they were soon lost in minute ramifications. The internal structure of the cyst much resembled that of the urinary bladder. There was neither outlet nor obvious communication with any other part. Several circumstances concurred to render the examination extremely hurried and unpleasant. The other cavities could not be inspected. A hasty outline of the external organs was with great difficulty sketched. In a case like this, the inutility of any attempt to discharge the fæces by an operation in the usual site of the anus, is at once obvious.

*Minutes of a dissection.* — Dr. Parry, in his Elements of Pathology, &c. page 187, remarks a frequent coincidence, whether as cause or effect, between enlargement of the thyroid gland and cardiac diseases. One of the Editors of this Journal has lately seen three cases of this complication. A lady died, a few months ago, at Ryde, in the Isle of Wight, after a lingering illness, exhibiting, among various anomalous symptoms, some strong signs of cardiac lesion. The following appearances presented themselves on dissection. "The thyroid gland was much enlarged; scirrhous, adhering to, and *pressing on*, the left carotid artery. (She had suffered under bronchocele for several years.) The body was much emaciated; the whole of the right hypochondriac region was livid. On removing the sternum, the lungs did not exhibit any thing particular, except some adhesions to the costal pleuræ. The pericardium adhered in these places to the substance of the heart, and contained, in its cavity, four ounces of a brownish serum. The heart itself was greatly enlarged, and thickened in its parietes, particularly the *left* ventricle, which was filled with coagulated blood. The aorta was enlarged as it issues from the ventricle. The carnea columnæ were almost as hard as cartilage, and several white specks of incipient ossification were dispersed over the surface of the heart. Looking down on the diaphragm, the liver was seen projecting that muscle nine or ten inches higher up than on the other side. The liver was greatly enlarged, and its natural structure much deranged. The mesenteric glands were also, many of them, enlarged and diseased. The stomach was natural; but the spleen was much enlarged, and there was an abscess in its substance, filled with dark-coloured pus. The kidnies were considerably enlarged, but there did not appear much deviation from the natural structure."—The history of this case is not sufficiently known to authorise any opinion relative to the *priority* of the cardiac or hepatic lesion; or whether the bronchocele preceded or followed the visceral derangements. Could the pressure on the carotid artery by the thyroid gland disturb the functions of the heart? What is the nature of, and how is produced, that uneasiness about the *throat*, which is so often complained of by patients labouring under cardiac disease?

*Abscess of the liver bursting into the lungs.*—"A gentleman, 45 years of age, remarkably healthy and strong, fell, about twelve months ago, from a ladder, and received a contusion in the right lumbar region, which he disregarded at the time, but which continued to occasion pain about the right kidney, with occasional depositions of sediment in the urine. In June last, however, a violent attack of acute hepatitis was with difficulty subdued by bleeding, purging, and mercurials. From that period to the beginning of September he looked sallow; lost flesh and strength; was constipated, and had frequent fits of bilious vomiting, the ejected matter resembling the yolks of raw eggs. A dull pain was complained of in the right side, and also in the shoulder; but he kept on to his business, and was seldom confined to the house. He consulted a physician in London of great eminence, who ordered him the blue pill with pulv. rhei every second night, and infusion of cascarilla with carbonate of ammonia in the day.

On the 9th of October, the pain in the right side became somewhat augmented, and the reporter advised both general and local abstractions of blood, with blisters and purgatives, of the mercurial kind. It was, however, too late, for the pain, fever, and disordered secretions increased till the 26th of October, when, with only a prelude of a few hours slight cough, an abscess suddenly burst into the lungs through the diaphragm, and in the course of a night, nearly a quart of ill-conditioned pus was discharged. From this time he lingered ten days; every thirty-six or forty hours having a fresh eruption of matter, after which the discharge would almost entirely cease for a period. Hectic fever, of course, went on all this time, and also pulmonic inflammation, from the pressure of the hepatic abscess first, and afterwards from the discharge of matter from the liver into the lungs.

The Reporter has no doubt, from much observation on these kinds of cases, that matter had been formed during the acute attack in June, and only produced the fatal catastrophe in November following. The fall was in all probability the original cause of all; and had timely evacuations been then had recourse to, the fatal train of symptoms which succeeded might possibly have been prevented."

We noticed in our last Number, the formation of a Society for granting annuities to Medical Practitioners who shall have attained the age of 60 years: the following is the scale of payments which will entitle the respective members of the Society to the benefits of the accumulated fund.

*TABLE shewing the Sum to be paid at one Payment, or the Sum to be paid Annually, in order to secure an Annuity of Fifty Pounds to the Subscriber during the remainder of his Life, after having attained the age of Sixty.*

| AGE. | SINGLE PAYMENT. | | ANNUAL PAYMENT. | | AGE. | SINGLE PAYMENT. | | ANNUAL PAYMENT. | |
|---|---|---|---|---|---|---|---|---|---|
| | £. | s. | £. | s. | | £ | s. | £. | s. |
| 23 | 43 | 18 | 2 | 19 | 37 | 96 | 16 | 8 | 4 |
| 24 | 46 | 8 | 3 | 3 | 38 | 102 | 14 | 8 | 18 |
| 25 | 49 | 0 | 3 | 8 | 39 | 109 | 0 | 9 | 15 |
| 26 | 52 | 0 | 3 | 12 | 40 | 115 | 10 | 10 | 11 |
| 27 | 55 | 10 | 3 | 18 | 41 | 122 | 16 | 11 | 13 |
| 28 | 58 | 0 | 4 | 3 | 42 | 130 | 12 | 12 | 16 |
| 29 | 61 | 4 | 4 | 9 | 43 | 138 | 18 | 14 | 3 |
| 30 | 65 | 0 | 4 | 16 | 44 | 147 | 18 | 15 | 11 |
| 31 | 68 | 16 | 5 | 5 | 45 | 157 | 8 | 17 | 8 |
| 32 | 74 | 14 | 5 | 15 | 46 | 167 | 16 | 19 | 5 |
| 33 | 76 | 16 | 6 | 1 | 47 | 178 | 18 | 21 | 15 |
| 34 | 81 | 6 | 6 | 9 | 48 | 190 | 18 | 24 | 3 |
| 35 | 88 | 10 | 6 | 19 | 49 | 203 | 16 | 27 | 17 |
| 36 | 91 | 6 | 7 | 10 | 50 | 217 | 16 | 31 | 11 |

As we have been informed that some Army and Naval Medical Gentlemen have taken legal advice relative to their security against the late Act for Examination at Apothecaries Hall, previously to settling in practice; and that they have been given to understand that the Act applies equally to them as to others; we think it right to give publicity to the circumstance, lest some of the said corps should be put to much inconvenience or loss from ignorance of the Act.

Thus it appears, that the first surgeons of our army and naval hospitals, must, if they desire to settle in general practice on their native soil, repair to Apothecaries Hall, there to undergo an examination by twelve apothecaries, "*To ascertain their skill and abilities in the science and practice of* MEDICINE!" Surely such indignities could never have been meditated by the legislature for those who treated our immortal wounded countrymen, at Trafalgar and Waterloo! Such, however, is the case, and the grievance cannot be too soon promulgated.

Previously to examination, each candidate must produce testimonials that he is twenty-one years of age; has served an apprenticeship; has a competent knowledge of Latin; has attended courses of lectures on anatomy, physiology, chemistry, materia medica, and the theory and practice of medicine; also of having attended a public hospital.

The actual examination consists — 1. In translating the Latin Pharmacopœia, and Physician's prescriptions. 2. Pharmaceutical Chemistry. 3. Materia Medica. 4. *Physiology.* 5. The Practice of Physic and *knowledge of diseases!* 6. Botany, next year.

Every person dispensing and prescribing medicines without the certificate of examination, "is liable to a penalty of *twenty pounds each time he visits or prescribes* medicine for the sick."

Not more than twenty have yet offered themselves as candidates at Apothecaries Hall, so great, it is supposed, is the dread of this awful tribunal; while, on two examining days, at Surgeons' Hall, 105 candidates passed and received diplomas!

A celebrated writer, in the Morning Chronicle, makes the following modest reflection on this circumstance. "It is evident the testimonials of eligibility, and the subjects of examination being different, that a candidate may pass there [Surgeons' Hall] with great eclat, who has not the requisite qualifications for the examination at Apothecaries' Hall." *Vide Medical Repository for January, p.* 88

Philo-Medicus asserts, that he knows that many students have imbibed the notion, that if they possess a surgeon's diploma, they may legally practice *physic*, without the Apothecaries' certificate. But this impression he declares is erroneous and dangerous.

The Editors of the Medical Repository have very judiciously thrown out a hint respecting two dilemmas not mentioned in the act, nor in Philo-Medicus's Letter, to which noncompliance with the act, will subject those entering into practice; namely, 1st. "that having no real designation or rights, any contract of copartnership with a legal Apothecary would be liable to be vitiated, at the pleasure of the partner practising before the passing of the act, or having a certificate. Second, Such pretended Apothecaries will have no exemption from sitting on juries, leets, or parochial offices."

*Medical School of St. Thomas's and Guy's Hospitals.* — The Spring Courses of Lectures at these adjoining Hospitals will commence the beginning of February, as follows, viz. — At St. Thomas's: Anatomy, and Operations of Surgery, by Mr. Astley Cooper and Mr. H. Cline. Principles and Practice of Surgery, by Mr. Astley Cooper. — At Guy's: Practice of Medicine, by Dr. Babington and Dr. Curry. Chemistry, by Dr. Babington, Dr. Marcet, and Mr. Allen. Experimental Philosophy, by Mr. Allen. Theory of Medicine, and Materia Medica, by Dr. Curry and Dr. Cholmeley. Midwifery, and Diseases of Women and Children, by Dr. Haighton. Physiology, or Laws of the Animal Œconomy, by Dr. Haighton. Structure and Diseases of the Teeth, by Mr. Fox. — N. B. These several Lectures are so arranged, that no two of them interfere in the hours of attendance; and the whole is calculated to form a complete Course of Medical and Chirurgical Instruction. Terms and other particulars may be learnt from Mr. Stocker, Apothecary to Guy's Hospital.

Dr. Merriman will re-commence his Lectures on Midwifery, at the Middlesex Hospital, on Thursday, February the 8th, at half past ten o'clock.

In a few days will be published an interesting Account of a Visit to London in the year 1814, exhibiting a comparative View of English and French Surgery, with some Observations relative to the London Hospitals: translated from the French of P. J. Roux, Professor of Anatomy and Surgery at Paris.

Nearly ready for publication, the Second Part of Orfila's System of Animal, Mineral, and Vegetable Poisons: this will complete the first volume. The two remaining Parts of this interesting Work will appear in the course of the ensuing Spring.

Dr. Armstrong, of Sunderland, has in the press a work, entitled "Practical Illustrations of Febrile Diseases." The principal object of this work is to shew the great advantages of early and decided blood-letting and purging in typhus, in a disease called the common inflammatory fever, in scarlatina maligna, in measles, in erysipelas, in hydrocephalus internus, in dysentery, and other disorders. It will be illustrated by several cases and dissections.

Dr. George Edward Male, Physician to the Birmingham Hospital, has in the press, and nearly ready for publication, in 8vo. "An Epitome of Judicial or Forensic Medicine, containing the Tests and Antidotes of Poisons; with Observations on Hanging, Drowning, Lunacy, Child-Murder, Abortion, &c. &c."

Dr. Stewart, Lecturer in Midwifery in London, has in the press, in 8vo. "Observations on Uterine Hæmorrhage."

Died.—John Norris, M. D. late of the island of St. Croix.—In Bedford Place, Russell Square, Sir Charles Blick, Surgeon.—Mr. Elliot, Apothecary to the City Dispensary.—In Baker Street, James Laird, M. D. formerly of the Bengal Medical Establishment.—Mr. Ed. Derby Lewis, Surgeon of H. M. S. Melville.—At an advanced age, Dr. Harrington, of Bath.

### METEOROLOGICAL REGISTER for NOVEMBER, 1815.

*Kept at Harwich, in Essex.*

By JOHN BAILEY, Surgeon, &c.

| Moon's Age. | | Atmospherical Pressure. Morn. | Ev. | Temp. | Wind. | Remarks. | General Statement of Diseases. |
|---|---|---|---|---|---|---|---|
| ● | 1 | 30 | 30 | 47 | N | Clear | |
| | 2 | 29 9 | 29 9½ | 47 | N | ditto | |
| | 3 | 30 1 | 30 1 | 42 | N | ditto | |
| | 4 | 2½ | 2½ | 39 | N | ditto | Ophthalmia |
| | 5 | 2 | 2 | 48 | NW | Misty | |
| | 6 | ½ | ½ | 47 | W | Fine | Pertussis |
| | 7 | ½ | ½ | 49 | W | Rain | |
| | 8 | | 29 9 | 41 | W | ditto | Intermittentes |
| ☾ | 9 | 29 7 | 9 | 53 | NW | ditto | |
| | 10 | 30 | 30 | 47 | W | | |
| | 11 | 29 8½ | 29 6 | 52 | SW | Rain | |
| | 12 | ½ | | 52 | SW | ditto | |
| | 13 | 1 | ½ | 54 | SW | Rain. G. of wind | |
| | 14 | 1 | ½ | 41 | W | Rain, viol. ſhower. | Windy |
| | 15 | | 1 | 35 | NW | Rain. G. of wind | |
| ○ | 16 | 2 | 4 | 32 | N | Ditto. Snow | |
| | 17 | 5½ | 7 | 30 | NW | Sharp froſt | |
| | 18 | 8 | 9 | 30 | NW | ditto | |
| | 19 | 30. | 30 | 29 | | Rain | |
| | 20 | | 29 6 | 45 | E | | |
| | 21 | 29 6½ | | 43 | E | Rain. Fog | |
| | 22 | 7½ | 7½ | 33 | NE | ditto | |
| ☽ | 23 | 30 | 30 1 | 32 | N | | |
| | 24 | 2 | 2 | 36 | N | | |
| | 25 | 3½ | 4½ | 47 | N | Foggy | |
| | 26 | 4½ | 3½ | 38 | N | Rain | |
| | 27 | 2½ | 1 | 41 | E | Ditto. Hail | |
| | 28 | 30 | 29 9 | 35 | N | | |
| | 29 | | 30 ½ | 30 | W | Foggy | |
| ● | 30 | 29 9 | 29 8 | 40 | S | Rain | |

During the greater part of this month ophthalmia has pretty generally prevailed: adults have been attacked by it with as much frequency as young children were in the preceding month; for the most part, the eyes have been afflicted in succession. In those who have had the diſeaſe the most severely, both eyes have been simultaneously affected. The free use of local blood-letting by leeches, with blisters frequently repeated to the neighbouring parts, accompanied likewise by soothing fomentations and collyria, have rescued several persons from the loss of viſion.—Intermittents have been general, and the majority of such patients have shewn an unusual tendency to hepatic disorder. Jaundice, so far as the observation of your Reporter goes, deserves not to be ranked among those complaints which are wafted about by atmospheric media; but at this part of our island, it has of late been more than usually frequent. Have those causes which develope the intermittent form of autumnal disease any share in producing this affection, separately and detached from any general disorder of the system?

### METEOROLOGICAL REGISTER FOR DECEMBER, 1815,

*Kept at Harwich, in Essex,*

By JOHN BAILEY, Surgeon, &c.

| Moon's | Age. | Atmospherical Preffure. Morn. | Ev. | Temp. | Wind. | Remarks. | General Statement of Difeafes. |
|---|---|---|---|---|---|---|---|
| | 1 | 29 8 | 29 9 | 45 | SW | Rain | |
| | 2 | 30 | 30 | 43 | W | | Ophthalmia |
| | 3 | 30 | 30 | 45 | SW | Rain, Foggy | |
| | 4 | 29 7 | 29 6 | 44 | W | Rain, G. of wind | Variola |
| | 5 | 8 | 3 | 41 | NW | Rain | |
| | 6 | 2½ | 3½ | 38 | N | Rain. G. of wind | Varicella |
| | 7 | 7 | 9 | 33 | E | ditto | |
| ☽ | 8 | 9 | 9 | 36 | E | ditto | Intermittentes |
| | 9 | 30 | 30 1 | 36 | E | | |
| | 10 | 2 | 2 | 33 | NE | Snow | Tonsilitis |
| | 11 | 2 | 2 | 32 | NE | | |
| | 12 | 1½ | 3 | 35 | NE | | Rheumatismus |
| | 13 | 1½ | 1½ | 34 | W | | acutus |
| | 14 | 1½ | 30 ½ | 33 | W | | |
| | 15 | 29 7 | 29 5 | 27 | W | Rain | Pneumonia |
| ○ | 16 | 28 9 | 28 8 | 42 | NW | ditto, G. of wind | |
| | 17 | 8 | 29 3 | 33 | NW | ditto with snow | Icterus |
| | 18 | 29 3 | 8 | 29 | W | Snow | |
| | 19 | 5 | 4 | 27 | W | ditto | Scabies |
| | 20 | 1½ | 29 | 45 | SW | Rain | |
| | 21 | 2 | 5 | 39 | W | | |
| | 22 | 5 | 7 | 31 | N | | |
| | 23 | 5 | 5 | 35 | W | | |
| | 24 | 2½ | 2½ | 39 | W | Rain | |
| | 25 | 4 | 7 | 26 | NW | | |
| | 26 | 5 | ½ | 36 | SW | Rain | |
| | 27 | 5 | 6 | 30 | NW | Rain, G. of wind | |
| | 28 | 8 | 7 | 31 | W | Rain | |
| | 29 | 7 | 7 | 47 | W | Gale of wind | |
| ● | 30 | 9 | 30 3 | 41 | NW | ditto | |
| | 31 | 30 3½ | ½ | 31 | SW | Rain | |

The last column of this Register exhibits a pretty copious supply for observation, and the frequency and violence with which most of the diseases there enumerated arose, furnished the Reporter with abundance of employment.

OPHTHALMIA. — This complaint, which shewed itself first amongst young children, attacked afterwards adult persons, and has hung about this town and neighbourhood for more than a year, as may be seen by reference to the Reports made through the medium of your Journal. Although not, generally, of that acute form which rapidly destroys the delicate structure of the part, yet it has, on almost all occasions, proved obstinate, painful, and of long continuance.

VARIOLA.—A few cases of this pestilence crept into the town, from our proximity and frequent intercourse with the metropolitan town of an adjoining county; where, although every effort has been made to arrest its progress, it has spread wide its baneful influence.

VARICELLA.—This simple but contagious complaint, which your Reporter has had occasion to observe on divers opportunities, both precedes and accompanies Variola, and very often is mistaken, by superficial observers, for a mild instance of that malady: a subsequent attack of small-pox removes the doubt. Its present visitation preceded that of Variola some weeks.

INTERMITTENTES—are not so frequent as usual, but are more difficult of removal.

TONSILITIS.—Several most severe attacks of this complaint have happened, attended by symptoms that demanded a prompt and vigorous mode of resistance.

RHEUMATISMUS ACUTUS.—The toilsome avocation of a mariner, the quiescence of his body after violent exertions, his exposure to a humid atmosphere, and the frequent interruptions to his hours of repose, make him peculiarly liable to this complaint. In the winter season, it is the principal part of Reporter's employment to combat its fugacious attacks. During the present and preceding months some very obstinate and long-continued cases have occurred.

PNEUMONIA—is of all other diseases the most frequent and the most fatal at this part of the kingdom during the winter season: cold north-westerly winds, veering about from that to an easterly quarter, are sure to bring in their train all the variety of pectoral and guttural derangement. Young and elderly subjects generally sink beneath violent attacks.

ICTERUS and SCABIES.—That sensible and accurate observer, Dr. Huxham, whose name has frequently been referred to in these Reports, noticed, in many years, that Itch was a disorder very rife in his neighbourhood, but offers no conjecture upon the subject. Other causes, besides those of an atmospheric kind, may explain the matter: the same observation applies to Jaundice, which, during this and the last month, has, in point of frequency, yielded to no other disorder. As the right learned Editor of Mr. Moore's Almanack says, in conclusion of his prophetic remarks, *we shall leave these matters for time and the curious to develope.*

